# Actinomyces meyeri Causing Cerebral Abscess in a Patient on Methotrexate: A Rare Case Report and Systematic Review of the Literature

**DOI:** 10.7759/cureus.41204

**Published:** 2023-06-30

**Authors:** Giulio Verrienti, Gianluigi Megliola, Emanuele Antonaci, Armando Gisotti, Cecilia Raccagni

**Affiliations:** 1 Neurorehabilitation, Casa di Cura Villa Verde, Lecce, ITA; 2 Anesthesia and Critical Care, Ospedale "L.Bonomo", Andria, ITA; 3 Neurology, Provincial Hospital, Bolzano, ITA; 4 Neurology, Lehrkrankenhaus der Paracelsus Medizinischen Privatuniversität, Bolzano, ITA

**Keywords:** anaerobic brain abscess, rheumatoid, oral methotrexate, actinomyces meyeri, immunocompromised brain abscess

## Abstract

Central nervous system (CNS) actinomycosis is a rare, serious, life-threatening, suppurative infection caused by Actinomyces species. Actinomyces are anaerobic Gram-positive bacteria, which can be normally isolated from the polymicrobial flora of the gastrointestinal- and genital tracts. They are considered very low virulent bacteria to humans. However, they can lead to several types of local or disseminated infections, if certain pathologic states or immunodeficiency occur. Intracranial abscesses caused by Actinomyces meyeri are rarely reported in adults. In this case report, we describe a 66-year-old woman who presented to the emergency department due to progressive complaints of altered sensorium and low-grade fever, due to an A. meyeri-related brain abscess. The only risk factor was represented by immunodeficiency due to the therapy with Methotrexate and steroids.

## Introduction

Actinomycosis is a rare infection caused by Actinomyces species. More than 40 species of Actinomyces have been recognized. Actinomyces are anaerobic Gram-positive bacteria, which normally colonize the oral cavity and the gastrointestinal and genital tracts. They can be isolated from the polymicrobial flora but, in some conditions, they may lead to the formation of local abscesses. The most common sites of localization of the infection are the cervicofacial area, followed by the pelvic region and the thorax. Diagnosis can be challenging and is supported by anamnesis, clinical and laboratory findings, and imaging. Several risk factors for actinomycosis, such as chronic alcohol abuse, diabetes, cancer, immunosuppressive therapy, organ transplant, and end-stage renal disease, have been encountered.

Central nervous system (CNS) actinomycosis is a rare suppurative infection that manifests itself by non-specific signs. In contrast to systemic actinomycosis, CNS actinomycosis is not usually associated with immunodeficiency [1], and in most cases, its pathogenetic mechanism is hematogenous spread or local extension of cervicofacial actinomycosis. The most common clinical presentation of CNS actinomycosis is an abscess (67%), followed by meningitis or meningoencephalitis (13%), actinomycoma (7%), subdural empyema (6%), and epidural abscess (6%) [1,2].

Here, we illustrate a case of a female patient on Methotrexate (MTX) and steroids, presenting with two cerebral abscesses caused by Actinomyces meyeri. She underwent drainage through craniotomy and was subsequently put on intravenous amoxicillin and clavulanate potassium.

## Case presentation

A 66-year-old woman was brought by her family to the emergency department (ED) with 6h history of altered sensorium and progressive inability to stand and walk normally. Mild fever and headache episodes in the previous week were reported by the relatives. Before admission, the patient was described as a fully autonomous person. Past medical history was significant for rheumatoid arthritis (RA) treated with MTX (12.5 mg s.c./week) and low dosis cortison during the last three years. At hospital admission, the patient was disoriented to time and place, but able to obey simple commands. The first physical examination in the ED was remarkable for a left hemiparesis (3/5 grade on the Medical Research Council Scale on both upper and lower limbs), hyperreflexia and positive upper motor neuron signs. The patient was unable to get up without assistance and to stand on her feet without falling on her left side. A decreased repetitive finger movements in her left upper extremity were also reported. Furthermore, the patient presented with mild dysarthria, while other cerebellar signs were unremarkable. Vital signs manifested with a temperature of 37.6 degrees Celsius, heart rate of 89 beats per minute, blood pressure of 140/90 mmHg. Blood tests on admission showed a raised C reactive protein of 68 mg/dL, white cell count 9.1×10^3^/µL and neutrophils 7.2×10^3^/µL. Chest x-ray was normal. Because of the neurological status cerebral imaging was performed.

**Figure 1:**
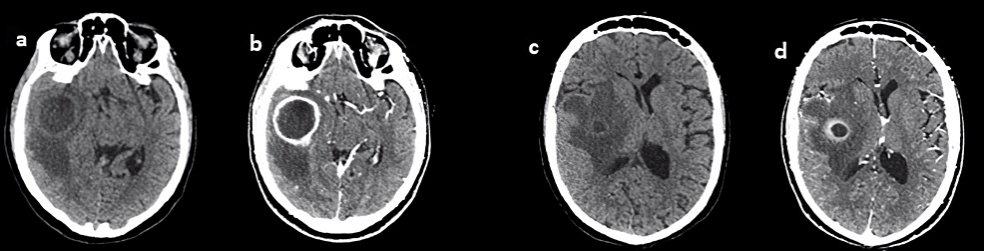
Initial brain computed tomography, pre- (a, c) and post (b, d) contrast, demonstrating two ring-enhancing lesions in the right temporal and parietal lobes.

Brain computed tomography (CT) revealed the presence in the right temporal lobe of “2 adjacent, rounded, hypodense lesions, the largest with about 4x4 cm in dimensions, bordered by large patches of perilesional edema”. An about 10 mm midline shift of the ventricular system was described. Thus, the subsequent administration of contrast demonstrated a ring-like enhancement of the identified lesions (Figures 1a-1d).

**Figure 2:**
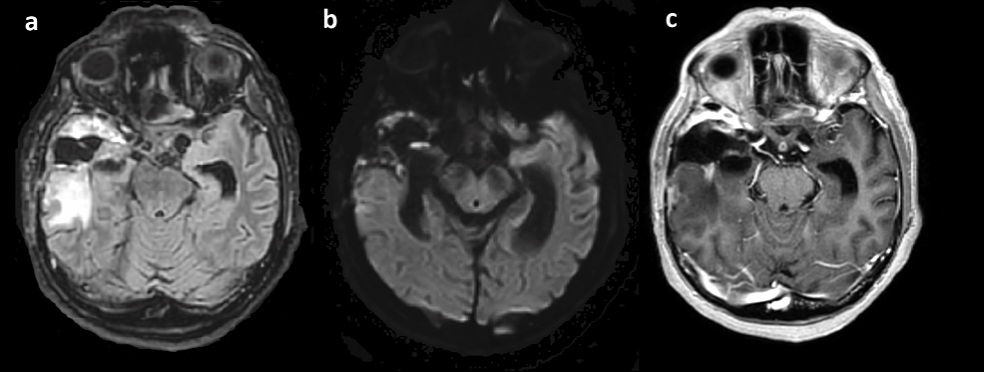
One-month follow-up brain magnetic resonance imaging (MRI) (a) Axial T2 weighted sequence showing neurosurgical residuals one month after abscess evacuation; (b) DWI-sb1000 and (c) axial contrast-enhanced 3D MPRAGE sequence showing no abscess residuals. DWI: Diffusion-weighted imaging; 3D MPRAGE: three-dimensional magnetization-prepared rapid gradient-echo

Based on the imaging aspects, the most probable diagnosis was cerebral abscess, even though CNS tumor or brain metastases were considered in the differential diagnosis. As the neurological condition worsened over few hours and the patient developed a state of unresponsiveness and respiratory distress, with emerging decerebration patterns, she was intubated and taken to the intensive care unit (ICU). An urgent, right-sided craniotomy was performed. Intraoperatively the initial supposed diagnosis of cerebral abscesses was confirmed. Drainage purulent abscess material underwent extended incubation. Through our automated instrument for microbial identification and antibiotic susceptibility (VITEK® 2 Compact, BioMérieux, Lyon-France) a metronidazole-resistant, but multi-antibiotic sensitive A. meyeri was identified with 94% accuracy. This result was confirmed through matrix-assisted laser desorption/ionization time-of-flight (MALDI-TOF) technology by an external labor service. A bacteremia could be excluded by several negative blood cultures. Based on the antibiogram of the evacuated abscess material, the patient was started on intravenous amoxicillin and clavulanate potassium. During the following days after neurosurgical treatment, the patient was difficult-to-wean off, thus a percutaneous tracheostomy was performed. At suspension of the sedation, the patient presented with an impaired consciousness and a left hemiplegia; her vigilance was reduced, but she was able to react adequately with eye closure to command. Voluntary, purposeful movements of the right limbs could be performed. In the following days the neurological conditions did not improve consistently. A percutaneous endoscopic gastrostomy (PEG) was placed. The patient remained in the ICU 16 days, thereafter she was discharged to the rehabilitation center, where she was started on intensive neurorehabilitation. A one-month follow-up cerebral magnetic resonance imaging (MRI) showed no abscesses residuals (Figures 2a-2c).

## Discussion

**Figure 3:**
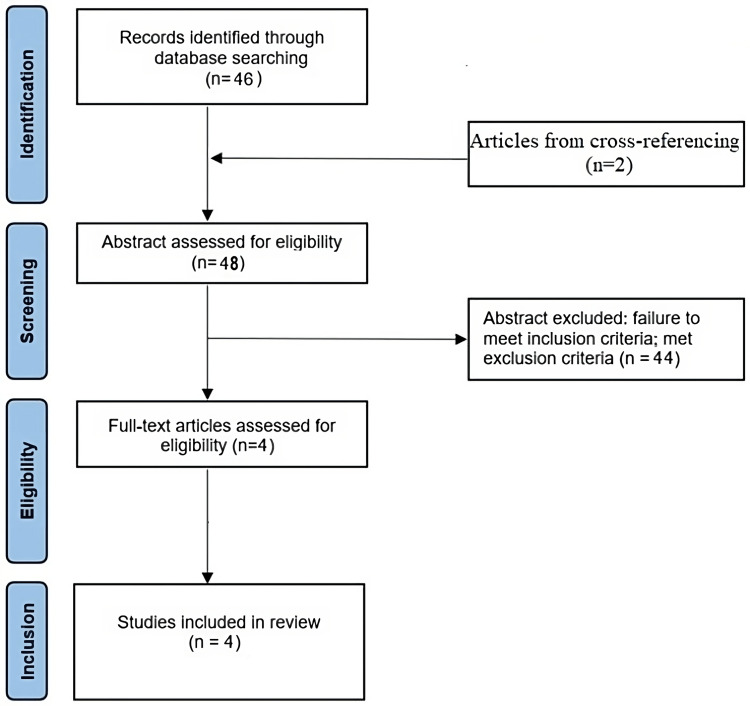
PRISMA flow chart for selection of articles for the systematic review. PRISMA: Preferred Reporting Items for Systematic Reviews and Meta-Analyses

First described in 1911 by Kurt F. Meyer, A. meyeri is an anaerobic, branching-filamentous Gram-positive bacillus. Infection by A. meyeri itself is uncommon, accounting for only 1% of all Actinomyces spp.. In contrast to A. israelii, which primarily causes cervicofacial infections, the most common site of infection by A. meyeri is the lung [2]. A peculiar issue of the infection caused by A. meyeri is its systemic dissemination. In this context, skin, long bones, liver and muscles are most frequently the target organs. The CNS is rarely affected by A. meyeri and usually presents as brain abscesses. In year 2017, Guillamet et al. [2] reported a case of a brain abscess caused by A. meyeri and identified seven other cases [3-8] in the literature. We performed a systematic review of the literature, following Preferred Reporting Items for Systematic Review and Meta-Analyses (PRISMA) guidelines. The PubMed database was searched in May 2023 with search terms “actinomyces” AND/OR “actinomycosis” AND/OR “actinomyces meyeri” AND “brain abscess” OR “cerebral abscess” AND “case report” AND/OR “case series” for articles published between January 2017 and April 2023. We included: (1) articles published in the English language; (2) case reports and/or case series regarding brain abscess induced by A. meyeri; (3) articles published in English in peer-reviewed journals; (4) studies conducted in the above-reported period. In the main search strategy, we excluded: (1) studies for which the complete text could not be found; and (2) articles not in English. The search yielded 46 articles; in addition, two articles were identified from cross-referencing. A total of 44 of these were excluded after assessing their abstract, as they did not meet the inclusion criteria. Four other case reports [9-12] of intracerebral abscesses induced by A. meyeri have been published since 2017 (Figure 3).

Results of this systematic review and description of the identified case reports are reported in Table 1.

**Table 1:** Description of the case reports included in the systematic review

Reference	First Author	Year of pubblication	Case description
[9]	Sah R	2020	A 35-year-old male patient who experienced an insidious headache and left-sided weakness. In this case, brain abscess was likely due to spread of Actinomyces meyeri from oral cavity to brain parenchyma. The patient underwent craniotomy was and the lesion was excised.
[10]	Pereira AJDSPR	2022	A 60-year-old man who experienced headache, barely perceptible speech and left-sided weakness. In this case, brain abscess caused by Actinomyces meyeri was proven to be of dental origin. The patient was submitted to surgical drainage.
[11]	Altdorfer A	2021	A 62-year-old male patient admitted to the emergency department after sudden onset of aphasia and confusion. In this case, a previous dental procedure could be identified as risk factor for the brain abscess caused by Actinomyces meyeri. The patient underwent drainage through craniotomy and was subsequently put on intravenous antibiotic therapy.
[12]	Funakoshi Y	2020	A 57-year-old woman initially presented with a 5-day history of headache, left arm numbness and weakness. A subdural abscess caused by Actinomyces meyeri associated with dental treatment was diagnosed. The patient underwent emergent navigation-guided drainage.

Adding the results of our review to the previous research of Guillamet et al. [2], we found that a total of 12 cases of brain abscesses induced by A. meyeri were published in the literature. Main features of these 12 case reports are summarized in Table 2. Anyway, the real incidence of brain abscess induced by A. meyeri may be underestimated due to the difficult diagnosis process and to the lack of notification obligation of actinomycosis.

**Table 2:** Main features of published case reports regarding brain abscesses induced by Actinomyces meyeri In year 2017, Guillamet et al. [2] presented a case report of a brain abscess caused by Actinomyces meyeri and identified seven other cases [3-8] in the literature. Four similar cases [9-12], included in our review, were published thereafter.

Author (reference), Year	Age Sex	Co- morbidities	Presumed Precipitant	Clinical presentation (time of onset; symptoms)	Imaging finding	Other cultured organisms	Neurosurgical approach	Antibiotic treatments	Outcome
Dijkmans [3], 1984	28, F	No significant past medical history	Unknown	3 days; Headache and aphasia	Left parietal lobe brain abscess, ventri-culitis	Streptobacillus moniliformis	Burr hole drainage, recurrent percutaneous punctures for external drainage	Benzathine penicillin x 2 m + dexamethasone x 1 m	No recurrence at 1 year
Park [4], 2014	46, M	No significant past medical history	Unknown	3 days; Headaches, fever, altered mental status, meningismus, right sided hemiparesis	Left lung mass, Left fronto-parietal lobe brain abscess	Propionibacterium Acnes, Fusobacterium nucleatum	Stereotactic brain biopsy	penicillin x 4 w, metronidazole x 4 w, amoxicillin x 11 m	No recurrence at 4 months
Clancy [5], 2015	55, F	History of six dental implants	Dental extraction 7d prior	Hours; altered mental status and new onset seizure	Left parietal lobe brain abscess	Group B streptococcus Staphilococcus capitis	Craniotomy and drainage	Vancomycin x 11 d, metronidazole IV x 1 m, Ceftriaxone x 4 m, amoxicillin x 6 m	No recurrence at 4 months
Fernandez-Valle [6], 2014	57, M	No significant past medical history	Dental extraction 7d prior	Hours; altered mental status and new onset seizure	Left parietal lobe brain abscess	Not reported	Stereotactic brain biopsy	Ceftriaxone and metronidazole IV x unknown duration, amoxicillin x 12 m	No recurrence at 1 year
Kuijper [7], 1992	44, M	Alcoholism	Unknown	1 month; R-sided weakness and dysarthria	Left fronto-parietal lobe abscesses (x2), Right occipital lobe abscess	Actinobacillus actinomycentemcomitans	Stereotactic brain biopsy and drainage	Amoxicillin 6 w, amoxicillin x 12 m	Clinical cure; follow-up period not specified
Rolfe [8], 2019	55, M	Not reported	Pneumonitis	Unknown	Brain abscess	Actinobacillus, mixed anaerobic flora	Brain biopsy	Ceftriaxone x 1 m, oral penicillin x 5 m	Lost to follow-up
Rolfe [8], 2019	44, M	Not reported	Sinusitis	Unknown	Bone and brain abscess	Microaerophilic streptococcus, Streptococcus mitis	Brain biopsy and drainage	Ceftriaxone x 1 m, oral penicillin x 5 m	Lost to follow-up
Guillamet [2], 2017	29, M	Unrepaired congenital heart disease	Dental extraction 7d prior	6 days; Complete left-sided body weakness, numbness, and focal convulsions of his left upper and lower extremities	Right parietal cerebral abscess	Streptococcus intermedius, and Parvimonas micra	stereotactic-guided neurosurgical drainage	Initially oral doxycycline and intravenous vancomycin and metronidazole, then oral doxycycline and oral metronidazole.	After 5 months no recurrence
Sah [9], 2020	35, M	No significant past medical history	Unknown	3 weeks; insidious onset of headache and left-sided weakness	Bi-lobed lesion in the right parietal lobe	Not reported	Craniotomy followed by abscess excision	Oral ampicillin–sulbactam for 12 months	Not reported
Pereira [10], 2022	60, M	Type 2 diabetes mellitus, glaucoma, treated gastric cancer, alcoholism	No previous dental procedure, but several oral septic foci were identified	1 week: headache, barely perceptible speech, left sided hemiparesis, partial seizures	Brain abscess	Fusobacterium nucleatum	Surgical drainage	Ceftriaxone 2g every 12 hours and clindamycin 600 mg every 6 hours, penicillin 24 MUI a day and metronidazole 500 mg every 8 hours, intravenously for 3 weeks and orally thereafter	After one month of hospitalisation, the patient was transferred to the residence area hospital
Altdorfer [11], 2021	62, M	No significant past medical history	Dental procedure a week prior	sudden onset of aphasia and confusion	Brain abscess	Aggregatibacter aphrophilus	Surgical drainage	Ceftriaxone	No recurrence at 3 months
Funakoshi [12], 2020	57, F	Hypertension, dental problems requiring tooth extractions, alcoholism	Dental procedure 2 months prior	5 days; left arm numbness and weakness	Sub-dural abscess	Fusobacterium nucleatum	Surgical drainage	Penicillin G iv and metronidazole	No recurrence occurred at 6 months6

As shown in Table 2, a presumed precipitant factor can be identified in almost half of the included cases. It is commonly recognized that some conditions, including congenital heart disease, diabetes, alcohol abuse, previous neurosurgery, dental procedure or head trauma, may promote the formation of brain abscesses. The use of corticosteroids or immunosuppressive conditions are other well-known risk factors.

To the best of our knowledge, this is the first case of a A. meyeri related brain abscess, where the only risk factor was represented by the immunodeficiency due to the therapy with MTX and steroids. As above mentioned, it should be noted that, in contrast to the common Actinomycosis imitators, such as Nocardia and tuberculosis, which also may cause cerebral pathology, CNS actinomycosis is not usually associated with immunodeficiency [1].

MTX is a pivotal drug for RA management and represents a first-line therapeutic choice in many other inflammatory and rheumatic diseases. Anyways, several, sometimes serious, side effects, including bone marrow suppression, pulmonary disease and liver fibrosis, have been associated to MTX-therapy [13]. Opportunistic infections under MTX therapy, which could present as brain abscesses, are also possible.

**Figure 4:**
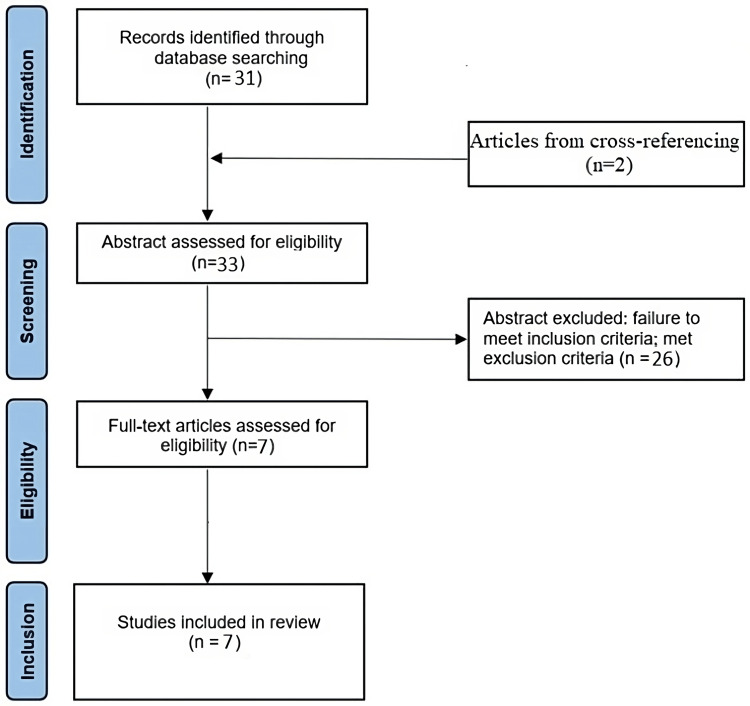
PRISMA flow chart for selection of articles for the systematic review. PRISMA: Preferred Reporting Items for Systematic Reviews and Meta-Analyses

According to PRISMA guidelines, we searched PubMed for case reports and case series published in English in peer-reviewed journals, using the MeSH terms “methotrexate, MTX, brain abscess, cerebral abscess.” The aim of this search was to quantify the incidence of brain abscess in rheumatic patients on MTX. The following exclusion criteria were adopted: (1) studies for which the complete text could not be found; (2) articles not in English; (3) oncologic cases; (4) cases in which other drugs with immunomodulating activity were administered. The search yielded 31 articles; in addition, a backward search (checking the bibliography of identified papers) found two other studies that were not retrieved using the main search strategy. A total of 26 of these were excluded after assessing their abstract, as they did not meet the inclusion criteria (Figure 4).

Thus, the literature review yielded seven case reports of patients (with different rheumatic diseases) on MTX with intracerebral abscesses, which we summarize in Table 3.

**Table 3:** Systematic review of case reports regarding brain abscesses in rheumatic patients on methotrexate According to PRISMA guidelines, we searched PubMed for case reports and case series published in English in peer-reviewed journals. The PubMed database was searched in May 2023 using the MeSH terms “methotrexate” OR “MTX” AND “brain abscess” AND/OR “cerebral abscess.” The inclusion criteria were (1) articles published in English language; (2) case reports and/or case series regarding brain abscess in patients on methotrexate; (3) articles published in English in peer-reviewed journals. In addition, a backward search (checking the bibliography of identified papers) was conducted to identify studies that were not retrieved using the main search strategy. The following exclusion criteria were adopted: (1) studies for which the complete text could not be found; (2) articles not in English; (3) oncologic cases; (4) cases in which other drugs with immunomodulating activity were administered. PRISMA: Preferred Reporting Items for Systematic Reviews and Meta-Analyses; RD: Rheumatic Disease; RA: Rheumatoid Arthritis CV: Cryoglobulinemic Vasculitis; NR: Not reported; AB: Antibiotic

Author (reference); year	Age, sex	RD	Identified microrganism	MTX therapy duration	Conco-mitant steroid therapy	Clinical presentation	CT/MRI findings	Treatment
Naqi R [14], 2011	73,M	RA	Nocardia Asteroides	More than 15 years	Yes	Generalized tonic clonic seizures, leucocytosis	A ring enhancing lesion with surrounding oedema in right posterior parietal cortex	Craniotomy with aspiration of abscess and long term AB-therapy (Sulfamethoxazole-Trimethoprim)
Ho CL [15], 2005	48, F	Neuro-Behcet	Coagulase negative staphylo-coccus	4 years	Yes	Generalised tonic seizure	A hyperintense lesion at the right parieto-occipital lobe surrounded by massive oedema	Osteoclastic trepanation and excision of the lesion followed by long term antibiotics and steroid therapy
Rafiei N [16], 2017	80,M	RA	Nocardia Farcinica	NR	Yes	Back abscess	Single Ring enhancing lesion in the frontal lobe	Drainage and long-term antibiotic therapy
Peterlana D [17], 2014	72,F	Horton’s arteritis	Listeria monocyte- genes	3 months	Yes	Left hemiplegia with ipsilateral hypoesthesia	Two lesions with ring-like enhancement located in the posterior limb of the internal capsule and pons	Long term AB therapy
Habib S [18], 2018	74, M	Psoriatic arthritis	Actinomyces viscosus	NR	NR	Generalized weakness and difficulty to ambulate	Scattered rim enhancing lesions with surrounding oedema throughout the brain, largest being 1.4 cm in the left frontal lobe	Long term (6 months) AB therapy (penicillin G 4 million units every four hours)
Lauerer RJ [19], 2021	66, F	CV and Sjögren’s syndrome	Scedosporium apospermium	2 years	Yes	Consciousness impairment	Multiple abscess formations in the left lentiform nucleus and the periventricular region with meningeal contrast enhancement in T1-Sequences	Long term Voriconazole therapy
Matsuura J [20], 2018	65, F	RA	Toxoplasma gondii	6 years	NR	sensory hypoesthesia on the right side of the face	Ring-enhanced signal at medulla oblongata to inferior cerebellar peduncle by contrast-enhanced T1-weighted MRI	sulfamethoxazole/trimethoprim (ST) mixture (trimethoprim: 360 mg/day) for 8 days. Discontinuation of methotrexate

With regard to the existing literature, intracerebral abscesses in rheumatic patients on MTX are extremely rare. Importantly, as shown in Table 3, in almost the totality of the reported cases, patients on MTX were taking concomitant steroid therapy, which alone may predispose to opportunistic infection.

There is only a few literature on virulence factors of Actinomyces species. The existing data suggest that they are not producing classical exotoxins and are probably able to evade the host immune clearance-surveillance in immuno-competent patients [1]. For this reason, patients on immune modulating drugs may be prone to develop infections at a higher risk. Of note, in a case report [19] of this review, a patient on MTX developed disseminated actinomycosis with lung and brain lesions caused by Actinomyces viscosus. We hypothesize that Actinomyces may show enhanced virulence in some conditions, such as the concomitant use of MTX and steroids.

## Conclusions

We report a case of cerebral actinomycosis in a patient on MTX with no other risk factors. To our knowledge, there are no other cases of A. meyeri-related cerebral abscess in patients on MTX reported in the literature. Further studies are needed to explore the pathogenic role of A. meyeri in cerebral abscess formation in immunocompromised patients.
